# The role of surgical treatment in isolated organ recurrence of esophageal cancer—a systematic review of the literature

**DOI:** 10.1186/s12957-018-1357-y

**Published:** 2018-03-14

**Authors:** Dimitrios Schizas, Ioannis I. Lazaridis, Demetrios Moris, Aikaterini Mastoraki, Lazaros-Dimitrios Lazaridis, Diamantis I. Tsilimigras, Nikolaos Charalampakis, Theodore Liakakos

**Affiliations:** 1 First Department of Surgery, Laikon General Hospital, National and Kapodistrian University of Athens, 17 Agiou Thoma Str., Goudi, 11527 Athens, Greece; 2 Department of Surgery, University Hospital Basel, Basel, Switzerland; 3 Fourth Department of Surgery, Attikon University Hospital, National and Kapodistrian University of Athens, Athens, Greece; 4 Hepatogastroenterology Unit, Second Department of Internal Medicine, Attikon University Hospital, National and Kapodistrian University of Athens, Athens, Greece; 5 School of Medicine, National and Kapodistrian University of Athens, Athens, Greece; 6 First Department of Medical Oncology, Henry Dunant Hospital Center, Athens, Greece

**Keywords:** Esophageal cancer, Isolated recurrence, Metastasis, Solitary lesions, Surgical management, Metastasectomy

## Abstract

**Background:**

Despite the improvements in the early detection and treatment of non-metastatic esophageal cancer, more than half of patients undergoing a curative treatment for esophageal cancer will develop recurrence within three years. The prognosis of these patients is poor. However, a wide range in overall survival has been reported, depending on the pattern of recurrence, and no optimal treatment strategy following recurrence has yet been uniformly accepted.

**Aim:**

In this article, we aimed to systematically review the literature for the role of surgical resection of metachronous distant metastasis following primary treatment of esophageal cancer. Furthermore, we discuss possible factors that could possibly predict which patients may benefit from a surgical approach. A comprehensive literature search was conducted in PubMed using combinations of keywords.

**Results:**

Patients with recurrence may benefit of a multimodality treatment. Regarding the isolated recurrence of esophageal cancer in solid visceral organs, operative intervention has been proposed as a treatment that may offer a survival benefit in an individual basis. No definitive conclusions regarding the potential survival advantage offered by the surgical treatment of solitary recurrent lesions can be drawn. However, recent improvements in surgical treatment and optimization of perioperative management guarantee an acceptable operative risk, making surgical resection of solitary recurrence lesions a considerable therapeutic option.

**Conclusions:**

It can be conferred from the available studies that the surgical treatment of isolated recurrence from esophageal cancer may offer a survival benefit for properly selected patients. Prospective, multicenter studies might be useful to gain a better insight into those factors that affect selection of patients to take benefit from an operative intervention.

## Background

Nearly half of all patients undergoing curative esophagectomy for esophageal cancer develop recurrence, and in approximately half of these patients, the recurrence appears within the first year postoperatively. According to the type of recurrence, the metachronous lesions are classified as locoregional, distant, and of mixed type. Distant recurrences include hematogenous metastasis within a solid organ, abdominal paraortic lymph node metastasis, and peritoneal metastasis [1]. Solid organs presenting distant recurrence are usually the lung, the liver, the brain, the kidneys, and the adrenal glands [2]. The prognosis of patients with locoregional recurrence tends to be better than of those with distant metastasis, while the mixed type has the poorest outcome [3].

Abate et al. showed that survival is considerably improved in patients undergoing therapy for their recurrence [4]. However, there is no consensus concerning the type of treatment to be followed in case of recurrence. Regarding the patients with isolated tumor recurrence, salvage therapeutic options include systemic chemotherapy, irradiation, surgical resection, or a combination of the above. Because of poor prognosis, only a few retrospective studies with small series of selected patients and several case reports showing the results of surgical treatment exist. Therefore, the benefit of surgical resection as a part of multimodality treatment to patients with isolated distant recurrence in solid organs is controversial. The aim of this study is to review the outcomes of surgical management of such lesions and to discover which patients’ characteristics may predict a better outcome after surgical resection.

## Methods

### Search strategy and data sources

The review was conducted in line with the Preferred Reporting Items for Systematic Reviews and Meta-Analysis (PRISMA) guidelines [5]. A study protocol was agreed by all authors. Identification of eligible studies was performed through search of PubMed (MEDLINE) database until 10 August 2017. The following algorithm was applied: “(surgery OR (surgical treatment) OR metastasectomy) AND ((esophageal or oesophageal) AND (cancer OR carcinoma)) AND (metastasis OR metastases OR recurrence).” Two independent reviewers (DS, IIL) screened the available literature, and discrepancies were resolved by team consensus. Finally, reference lists of eligible studies were manually assessed in order to detect any potential relevant article (“snowball” procedure).

### Inclusion and exclusion criteria

Eligible were considered those studies reporting on patients undergoing surgery for metachronous, solitary organ recurrence following primary surgical treatment of esophageal cancer. Neither language nor study sample size restriction was applied. Exclusion criteria were as follows: (1) irrelevant studies, (2) studies reporting on synchronous metastases, (3) reviews and meta-analyses, and (4) editorials and letters to the editors.

### Data extraction and tabulation

Two independent authors (DS, IIL) extracted the data. Results were cross-checked by a third reviewer (DIT), and any discrepancies were resolved by team consensus. Variables of interest included general study characteristics (e.g., author, year of publication, number of patients), number of lesions, disease-free interval (DFI) following esophagectomy, type of operation, and survival following resection of recurrence. DFI was defined as the time from primary surgery for esophageal cancer to disease recurrence. Data were tabulated when possible. Due to the nature of the included studies (i.e., case reports) and the high heterogeneity in reporting of outcomes among eligible studies, no cumulative statistical analysis or meta-analysis was attempted. Ultimately, a purely descriptive presentation of available data was adopted.

## Results

### Article selection and study demographics

**Fig. 1:**
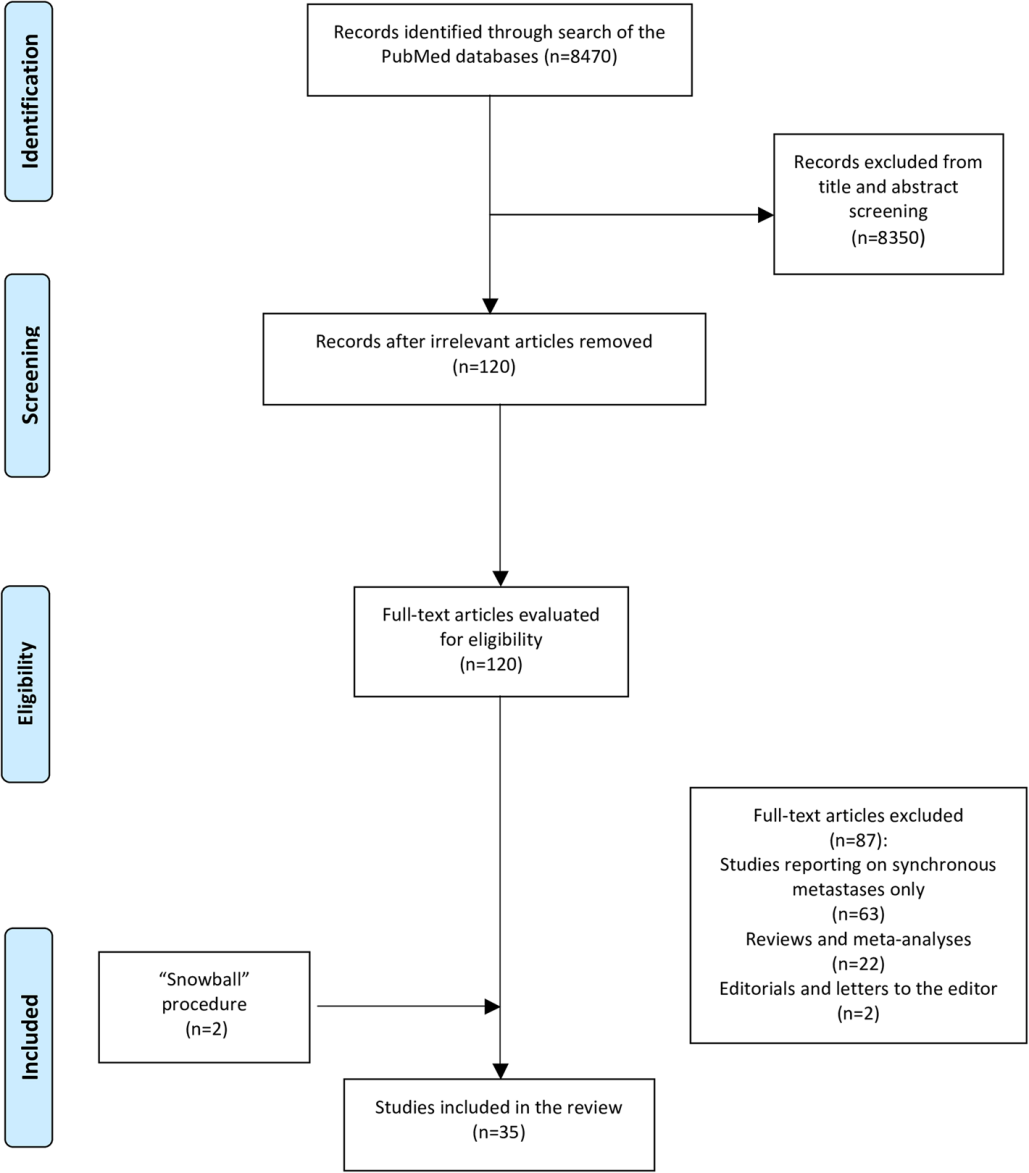
Flowchart of the search strategy

The results yielded by the initial algorithm and the successive steps of the selection process are depicted in Fig. 1. Following screening of titles and abstracts, 120 studies were retrieved for full-text evaluation. Thirty-three studies were deemed eligible, while two were identified from their reference lists for a total of 35 studies included in the analytic cohort. Overall, studies reported on isolated liver recurrences undergoing surgical resection (*n* = 87 patients), isolated lung (*n* = 163 patients), brain (*n* = 30), and other recurrences (*n* = 10 patients), encompassing a total of 290 patients included in this review.

### Liver

Liver represents a common organ for metastasis for a wide range of tumors. The benefit of liver resection for colorectal and neuroendocrine metastases has been proven and is considered as a standard treatment with curative intention when feasible. Few retrospective studies with > 50 highly selected patients aimed to show the benefit of liver resection for non-colorectal and non-neuroendocrine tumors. In most of them, however, the operative management for metachronous metastases of esophageal cancer represents only a very small part of the mentioned series. Adam et al. reviewed 1452 patients operated for non-colorectal and non-neuroendocrine liver metastases. Among them, 20 patients with liver metastases from esophageal cancer and 25 patients with liver metastases from gastroesophageal junction cancer underwent liver resection, reporting a 3-year survival of 32% and 12%, respectively and a median survival of 16 and 14 months, respectively [6].

Other studies focus on the outcomes of surgical management of liver recurrence after esophageal resection for both types of squamous cell carcinoma (SCC) and adenocarcinoma. The largest series is reported from Liu et al. in which 26 patients with solitary hepatic metastasis after esophagectomy for SCC underwent liver resection. The surgical group presents 1- and 2-year cumulative survival rates of 50.8 and 21.2%, respectively, which were significantly higher than the 31.0 and 7.1% survival rates of those patients in the non-surgical group (*n* = 43). In both groups, a DFI of more than 12 months after esophagectomy was connected to better outcomes [7]. Ichida et al. [8] reported also a group of 5 patients undergoing hepatectomy for liver metastases of esophageal cancer, who showed a median survival of 13 months following recurrence detection, compared to a median survival time of 5 months of the non-surgical group [8]. In a case series of 4 patients, Huddy et al. included two young patients of 44 and 47 years old, presenting with one and two liver metastases, respectively, 9 months after esophagectomy for adenocarcinoma. Both were treated with systematic chemotherapy and subsequent liver resection; the first one remained free of disease during the 23 months of follow-up, and the second one had an overall survival of 21 months following liver resection [9]. The outcomes of the abovementioned studies as well as of case reports of patients undergoing liver resection for the treatment of liver metastases from esophageal primaries described in the literature are summarized in Table 1 [6–16].

**Table 1:** Reported cases of surgical management of liver recurrence for esophageal cancer

Author (year)	Disease-free interval following esophagectomy (months)	Number of patients	Number of lesions	Operation	Survival following recurrence resection
Liu et al. 2016	14.15 ± 9.68^†^	26	1	2 left hemihepatectomies, 2 left lateral lobectomies, 2 right hemihepatectomies, 1 right anterior lobectomy, 19 non-anatomical hepatectomies	1- and 2-year cumulative survival rates of 50.8 and 21.2%, respectively
Hiyoshi et al 2015	12.2	1	N/S^‡^ (solitary or occurred in a localized field)	Partial hepatectomy	23.1 months
Huddy et al. 2015	34.5 (19–55)^§^	4	1–3	Resection segments 2 and 3, resection segment 5, right hemihepatectomy and extension to segment 4A, central hepatectomy with extension to segment 6	10 months, 21 months, 22 months alive, 92 months alive
Tada et al. 2015 (published in Japanese)	6	1	1	Right hepatic lobectomy	10 months alive
Blom et al. 2013	72	1	1	Hemihepatectomy	N/S^‡^
Iitaka et al. 2013	12	1	1	Partial metastasectomy	N/S^‡^
Ichida et al. 2012	6 (0–14) ^§^	5	1–3	N/S^‡^	13 months (2–70)^§^
Ikebe et al. 2012 (published in Japanese)	10	1	N/S^‡^	N/S^‡^	4 years alive
Tokairin et al. 2009 (published in Japanese)	19	1	1	S8-semi-segmental lobectomy, followed by right hepatic lobectomy and lymphadenectomy	N/S^‡^
Adam et al. 2006	N/S^‡^	45	N/S^‡^	N/S^‡^	16 months for esophageal primaries and 14 months for primaries of the gastroesophageal junction^§^
Nagano et al. 2001 (published in Japanese)	15	1	Multiple	N/S^‡^	2 years and 3 months alive

^†^Mean and standard deviation

^‡^Not stated

^§^Median and range

### Lung

Surgical resection is accepted as a treatment modality for resectable pulmonary metastases arising from various solid tumors, in the absence of other extrapulmonary metastasis. Several retrospective studies have intended to investigate its benefit in the case of metachronous, pulmonary metastasis after surgical treatment of esophageal cancer. Ichikawa et al. reported a retrospective series of 23 patients with pulmonary oligometastatic disease after curative treatment of esophageal cancer (esophagectomy ± neoadjuvant or adjuvant chemotherapy or definitive chemoradiotherapy). The lungs were the initial recurrence site in 19 patients. All patients underwent surgical resection, including wedge resection, segmentectomy, and lobectomy, and the predicted overall 5-year survival rate was 43.5% with a median survival time of 28.7 months (range 4.9–214.5 months). The authors suggested that the group of patients with pulmonary metastasis as initial recurrence had a significantly longer survival (median survival 63 months) in comparison to the patients with antecedent extrapulmonary metastasis (11 months, *p* = 0.0411) [17]. Kobayashi et al. reviewed 30 pulmonary metastasectomies of 23 patients who presented pulmonary recurrence after definitive treatment of SCC (17 patients underwent esophagectomy, 5 patients definitive chemoradiotherapy, and 1 patient endoscopic submucosal dissection). Among these surgical resections, 26 concerned solitary metastasis including recurring metastases, 2 were undertaken at patients with two metastatic lesions, and 2 at patients with three metastases. The cumulative survival for patients without a history of extrapulmonary metastasis between initial treatment and pulmonal recurrence was 40.7% at 5 years, while the patients with well and moderately differentiated SCC had significantly prolonged survival compared to those with poor differentiated primaries (*p* < 0.01). DFI of > 12 months was showed as another favorable prognostic factor (median survival with DFI > 12 months and DFI < 12 months was 43.7 and 24.7 months, respectively, *p* = 0.02) [18]. The largest published series is the one reported by Shiono et al., including 49 patients who underwent metastasectomy (48 cases with SCC, 1 case with adenocarcinoma), 39 of whom with solitary metastases. The authors identified DFI of less than 12 months to be an independent prognostic factor (*p* = 0.04) for significantly worse prognosis, thus concluding that pulmonary metastasectomy for esophageal cancer should be considered for selected patients with a DFI of 12 months or more [19]. Kanamori et al. published recently a series of 33 patients undergoing pulmonary metastasectomy, 27 of whom with single pulmonary lesions and 22 of them having the lungs as first recurrence site. The overall median survival time was 17.9 months. Significant unfavorable prognostic factors identified with univariate analysis were DFI < 16 months and nodal status of esophageal carcinoma, but multivariate analysis failed to identify these factors to be significant. Again, in this study, most primary tumors histology was SCC [20].

The differential diagnosis between metachronous metastasis of esophageal SCC and second primary lung SCC based only on histologic features can be a diagnostic challenge. Kozu et al. excluded in their retrospective study patients with DFI longer than 24 months, solitary lung lesions, and no signs of esophageal cancer recurrence after pulmonary resection because these clinical criteria may imply a second primary lung SCC. Based on these exclusion criteria, they included 15 patients who underwent pulmonary metastasectomy for pulmonary recurrence of esophageal cancer in their institution. The results showed a 3-year overall survival of 44%. The diameter of the pulmonary metastasis (more or less than 20 mm) was marginally associated with the overall survival (OS) (*p* = 0.087), but none of the parameters examined (age, tumor stage, DFI, CEA levels, surgical approach, and procedure) was found to be a significant prognostic factor [21]. Table 2 shows the results of studies concerning lung metastasectomy for esophageal carcinoma [8, 10, 17–24].

**Table 2:** Reported cases of surgical management of lung recurrence for esophageal cancer

Author (year)	Disease-free interval following esophagectomy (months)	Number of patients	Number of lesions	Operation	Survival following recurrence resection
Kanamori et al. 2017	15.5 (3–60)	33	27 patients with solitary tumors, 2 patients with 2 tumors, 4 patients with 3 or more	20 wedge resections, 6 segmentectomies, 7 lobectomies	17.9 (2–92)
Hiyoshi et al. 2015	26.4 (4.6–41.7)	4	N/S^†^ (solitary or occurred in a localized field)	4 partial pulmonary resections, 1 partial pulmonary resection with chest well resection, 1 bilateral pulmonary resection with chest wall resection	25.3 (10.7–30.4)
Kozu et al. 2015	15 (7–36)	15	1–2	11 wedge resections, 2 segmentectomies, 2 lobectomies	32
Kosaka et al. 2014	28	1	1	Thoracoscopic partial lung resection	12 months alive
Kobayashi et al. 2014	23.8 (0–61)	23	1–3	25 wedge resections, 2 segmentectomies, 2 lobectomies	37.4 (1–114)
Ichida et al. 2013	6 (0–18)	5	1–3	N/S^†^	48 (10–63)
Takemura et al. 2012	N/S^†^	5	1	5 wedge resections	48 (6–124) at the time of follow-up
Ichikawa et al. 2011	15.5 (3.8–79.1)	23	1–4	17 wedge resections, 3 segmentectomies, 3 lobectomies	28.7 (4.9–214.5)
Shiono et al. 2008	14 (0–124)	49	1–5	23 wedge resections, 16 lobectomies, 8 segmentectomies, 2 bilobectomies	27
Chen et al. 2008	21 (13–69)	5	1–5	2 wedge resections, 3 segmentectomies	24 (11–90) at the follow-up

Results are expressed as the median and range or the number

^†^Not stated

### Brain

The incidence of brain metastasis from esophageal carcinoma reported in the literature is 0–2% [25].The prognosis of these patients is poor, and a median survival of 3.8 months after the diagnosis of brain metastases has been reported [26]. Brain recurrences tend to occur in patients with large primary tumors and findings of local invasion and lymph node metastasis by CT scan and/or microscopic examination [27]. Moreover, in contrary to other primary tumors, a low incidence of lung metastasis at the time brain metastasis appears has been reported in patients with brain metastases from esophageal carcinoma [28]. In a single-center retrospective study, the outcome of surgical resection followed by whole-brain radiotherapy (WBRT) for brain metastases from esophageal carcinoma has been compared to radiation or palliative treatment. Five out of 26 patients with brain metastases from esophageal carcinoma underwent surgery followed by WBRT. Three patients presented a single cerebral lesion and 2 patients had 2 lesions. The median survival of these patients was 7.0 months, while patients who underwent radiation therapy and chemotherapy survived for 4.0 and 1.8 months, respectively. The authors concluded that surgical intervention, the presence of a single lesion, Karnofsky Performance Status (KPS), and extracranial disease status had a statistically significant impact on survival. However, the median time from the diagnosis of esophageal carcinoma to the diagnosis of brain metastasis was 10.2 months (0.0–39.2 months), indicating that patients with synchronous metastases undergoing palliative therapy have been included. Furthermore, no data about the initial treatment of the primary tumor are mentioned [29]. Ogawa et al. reported the outcomes of 36 patients with brain metastases from esophageal cancer treated in a single institution, 8 of whom had no active extracranial disease and controlled primary tumor (primary tumor in complete remission after surgical resection, radical radiotherapy/radiochemotherapy, or a combination therapy). Seventeen patients had a single cerebral metastasis. The median survival was 9.6 months for patients who underwent surgery and radiotherapy and 1.8 months for patients treated only with radiotherapy. Treatment modality, KPS, and extracranial disease status had a statistically significant impact on survival [28]. In another series of 27 patients with brain metastases (13 patients had a single brain lesion), the longest survival was seen in patients with single brain lesions who underwent resection followed by WBRT (median survival, 9.6 months; *P* = 0.02 compared with all other treatments). The median time from diagnosis of primary tumor was 5.6 months (range 0.0–36.5 months), and 19 patients suffered from systemic metastasis as well [26]. The most recent case series was reported in 2007. Out of 17 patients treated for brain metastasis (median time from diagnosis of primary tumor 12.3 months, range 2.1–36.2 months), 3 patients treated with resection and WBRT had a median survival of 65.6 months (range 2.3–90.6 months), and in all treatment categories, patients with single cerebral metastasis had a better outcome than those with multiple metastases (median survival 38.2 and 16.4 months, respectively). Again, in that case, authors do not mention what kind of therapeutic regime was followed for the primary tumor, but as authors of previous studies, they conclude that an aggressive strategy with neurosurgery followed by radiation offers favorable results in patients with good KPS [30].

### Other organs

The adrenal glands are a frequent site of recurrence of esophageal neoplasms: esophagus is the third most frequent site of origin of adrenal metastases. Although adrenalectomy is a clear indication for the treatment of adrenal metastases from some cancers (lung cancer, renal cell cancer, and melanoma), there are very few case reports of adrenalectomy for recurrence of esophageal cancer following esophagectomy in the literature and thus there are no clear indications on how to treat patients with adrenal metastases from esophageal cancers. In a case series of 5 patients, only one 79-year-old patient presented with no signs of other metastasis and was treated, 13 months after esophagectomy for a Siewert type 2 carcinoma of the esophagogastric junction, with a laparoscopic left adrenalectomy. He died with progressive disease 28 months later [31]. O’Sullivan et al. reported a case of a 50-year-old man who was submitted to an open right adrenalectomy 4 years after a two-stage esophagectomy for a Siewert type 1 cancer of the esophagogastric junction and remained disease-free for over 4 years postadrenalectomy [32]. In another case, a 71-year-old patient underwent a right adrenalectomy for a solitary adrenal metastasis that appeared 22 months after a subtotal esophagectomy with two-field lymphadenectomy for esophageal adenocarcinoma. The patient was free of recurrence for the next 5 years and 11 months [33].

The kidney is the fourth or fifth most common visceral metastatic site for a primary esophageal carcinoma, but a solitary, unilateral metastasis is rare and often found accidentally, since the patients are mostly asymptomatic. There are only very few case reports of surgical resection of renal recurrence of esophageal cancer after primary surgical treatment. Lim et al. reported a case of solitary recurrence 2 years after an esophagectomy for a SCC, which was treated with radical nephrectomy. The follow-up revealed suspicious paraaortic lymphadenopathy that decreased in extent after admission of palliative chemotherapy, but no data concerning the survival are mentioned [34]. A 57-year-old patient, treated with radiation therapy for esophageal cancer, showed a recurrence in the right kidney accompanied with a tumor thrombus in the inferior vena cava 1 year after. He was treated with right nephrectomy with thrombus resection and died 2 months later [35]. Sun et al. reported also a case of nephrectomy for a solitary renal metastasis 9 months after an esophagectomy for a SCC (stage pT2N0M0), and the patient died 3 months later [36].

Esophageal carcinoma is a rare cause of splenic metastasis, with only five reported cases of isolated splenic secondaries. Sanyal et al. reported the unusual case of a 25-year-old female patient who underwent splenectomy, distal pancreatectomy, and resection of the splenic colonic flexure for a 6 × 6 cm symptomatic solitary splenic recurrence 15 months after a transhiatal esophagectomy and adjuvant chemoradiotherapy for a SCC of the lower third of esophagus. She remained free of disease 7 months postsplenectomy [37]. Table 3 summarizes all case reports of the literature for metastasectomies undertaken for adrenal, renal, and splenic recurrences of esophageal cancer [31–40].

**Table 3:** Reported cases of resected solitary recurrence from esophageal cancer in other sites

Author and year	Recurrence site	Initial TNM stage	Disease-free interval following esophagectomy	Overall survival following recurrence resection
Sun et al. 2014	Kidney	T2N0M0	9 months	3 months
O’Sullivan et al. 2013	Adrenal glands	T3N0M0	4 years	4 years alive
Fumagalli et al. 2010	Adrenal glands	T2bN1M0	13 months	12 months
Cho et al. 2007	Adrenal glands	T2N1M0	8 months	42 months alive
Sanyal et al. 2005	Spleen	T3N1Mx	15 months	7 months alive
Lim et al. 2004	Kidney	N/S^†^	25 months	N/S^†^
Saito et al. 2010 (published in Japanese)	Adrenal glands	N/S^†^	1 year and 10 months	5 years and 11 months
Hata et al. 2000 (published in Japanese)	Adrenal glands	N/S^†^	8 months	14 months alive
Miyoshi et al. 1997	Kidney	N/S^†^	1 year	2 months
Shimada et al. 1992 (published in Japanese)	Adrenal glands	N/S^†^	4 months	18 months alive

^†^Not stated

## Discussion

Our study showed that there are only sporadic cases of surgical treatment of distant recurrence of esophageal cancer in visceral organs. The few case series and comparative studies include highly selected patients and are therefore subjected to selection bias. Large-scale randomized multicenter trials are unlikely to be feasible. Hiyoshi et al. reported a study comparing surgical and non-surgical treatment of distant recurrence. Among 14 patients that underwent surgical treatment, 6 patients underwent partial pulmonary resection, 1 patient underwent partial hepatectomy, and 1 patient resection of brain recurrence. The surgery group showed a more favorable prognosis in terms of both survival after esophagectomy and survival after initial recurrence [10]. The largest retrospective study comparing different treatment options for different subtypes of recurrence of esophageal cancer following curative surgical resection (anastomotic, locoregional, single solid organ metastasis, single metastasis at another location, multiple hematogenic metastasis, or mixed-type recurrence) has been conducted by Depypere et al. Regarding the subgroup of patients with single solid organ metastasis (liver, brain, lung, and adrenal), the authors found that the surgically treated patients, with or without systematic chemotherapy (*n* = 20), had a significantly better survival in comparison with the non-surgically treated patients (*n* = 63), with a median survival after diagnosis of recurrence of 54.8 months (5-year survival of 43.9%) and 11.6 months (5-year survival of 4.6%), respectively (*p* = 0.0004) [41]. However, in comparative studies, various prognostic variables are unevenly distributed among the surgical and non-surgical groups. For that reason, no definitive conclusions regarding the potential survival advantage offered by the surgical treatment of solitary recurrent lesions can be drawn.

However, recent improvements in surgical treatment and optimization of perioperative management guarantee an acceptable operative risk, making surgical resection of solitary recurrence lesions a considerable therapeutic option. Indeed, evidence shows that patients with a single distant recurrence may have a favorable prognosis in comparison to patients with more than one lesions [8, 20]. Taking into account, though the very small number of patients operated with more than one distant metastases, no statistical significance can be noted. Therefore, the choice of treatment modality should be individualized. Certain characteristics of patients with good functional status may indicate which patients could be surgical candidates in case of a technically resectable solitary distant recurrence. Moreover, since metastasectomy is widely accepted as a possible curative treatment modality for recurrences of cancers of various visceral organs, including colorectal cancer, conclusions drawn from the analysis of these primaries could be helpful in the comprehension of the benefit of surgical therapy in the esophageal cancer recurrence. Metastasectomy has been also applied as part of the treatment of highly selected patients with oligometastatic gastric cancer and some retrospective, non-randomized studies suggest that surgical intervention may prolong survival [42–44].

Other uncommon sites of isolated esophageal cancer recurrence include skin, eyes, muscle, heart, jaw, skull, breast, thyroid glands, and gastrointestinal tract. Special anatomic features of the esophagus, such as the absence of serosa, its shared arterial and venous vasculature, and its complex lymphatic drainage may be implicated in this rare distribution pattern of tumor recurrences. A systematic review of the literature of the past four decades has shown that surgical resection has been a part of the management in 44% of these special cases, which presented an overall survival rate of 13 and 6.1 months for synchronous and metachronous metastases, respectively [45].

DFI between the initial surgical treatment of the primary and the diagnosis of recurrence appears to be an important factor to be considered. Long DFI implicates a less aggressive tumor biology, and in case of a surgical excision of a distant recurrence, a local control of the disease could be possibly better achieved, potentially offering curative treatment. Most patients included in the abovementioned studies and case reports underwent a surgical resection after a DFI of more than 12 months. As discussed before, short DFI is negatively correlated with survival [20], and long DFI is considered as a favorable prognostic factor for overall survival of both surgical and non-surgical groups [7]. Kobayashi et al. and Shiono et al. suggested a DFI > 12 months as a statistically significant favorable prognostic factor for pulmonary metastasectomy (*p* < 0.05) [18, 19]. With regard to pulmonary recurrence, long DFI is noted to be a favorable prognostic factor from the International Registry of Lung Metastases, but many different tumor types have been assessed in this study [46]. Regarding surgical resection of liver metastasis of colorectal primary, there is no consensus regarding the impact of DFI on outcomes. Some authors have reported that a short DFI did not impact disease-free or overall survival; however, other investigators consider DFI as a reliable prognostic factor [47, 48].

Primary tumor stage has been also accepted as a significant prognostic factor regarding survival, with advanced tumor stage being associated with a worse survival. Increased depth of tumor invasion and the presence and the mean number of positive nodal metastases are found to correlate with an increased incidence of recurrent disease [2]. Regarding resection of colorectal liver recurrence, positive lymph node status is found to correlate with worse outcome. Between all studies regarding esophageal cancer recurrence mentioned above, only that of Kanamori et al. revealed primary positive nodal status as a significant unfavorable prognostic factor concerning survival after pulmonary metastasectomy [20]. Depth of invasion of the primary tumor could not be identified as an independent prognostic factor. Apparently, accumulation of more patients is again needed to evaluate the significance of these factors.

The size of metastasis is another factor under investigation for its significance regarding survival. In their study [8], included 138 patients with liver and/or lung recurrence after esophagectomy. The statistical analysis resulted in the identification of the maximum size of metastases as a predictor of survival in patients with hepatic and/or pulmonary metastases (risk ratio of 2.39, 95% 1.10–5.18 for maximum size of metastases ≥ 21 mm, *p* = 0.029), but the outcome refers to both surgical and non-surgical groups [8]. The abovementioned studies failed to show any significance of the size of distant recurrence for the survival benefit of the surgical resection.

The predominant histologic type of the cases mentioned above is the squamous cell carcinoma whereas the adenocarcinoma represents only a small percentage of all cases. Regarding the histological differentiation, only one study revealed poor differentiation as a prognostic factor influencing prognosis in patients undergoing pulmonary metastasectomy [18].

The site of the isolated distant recurrence appears to correlate with the location of the primary tumor; tumors located in the cervical and upper thoracic esophagus tend to recur more often in the lungs, while isolated recurrences of the tumors of the lower esophagus tend to appear mostly in the liver [8]. However, no conclusions regarding correlation of the primary tumor location and survival after recurrence resection can be drawn.

Other parameters investigated, such as age, gender, elevated tumor markers, initial curative treatment (esophagectomy or definitive chemoradiotherapy), or the operative procedure followed for the recurrence failed to show any relevance in terms of benefit of surgical resection.

Limitations of the studies mentioned above include their retrospective character, the relatively small number of patients included, leading to non-significant statistically conclusions, the selection bias due to the fact that patients with poor medical condition were generally excluded from any surgical treatment and the heterogeneity of the baseline characteristics of the study populations. Due to the lack of strict guidelines pertaining to the therapeutic approach in each recurrence site, well-organized prospective multicenter studies may offer a possibility to draw firmer conclusions. To this end, we suggest that—when feasible—future prospective studies should randomize patients with solitary distant recurrence and DFI > 12 months after curative esophageal cancer resection into surgery and non-surgery groups so that accumulating evidence can permit the formulation of strict guidelines in each setting in the future.

## Conclusion

Patients with isolated distant hematogenous recurrence represent a small subgroup of patients with recurrence after curative treatment of esophageal cancer. Multimodality treatment may improve the prognosis of this patient population. Surgical resection of these lesions as a part of this treatment may offer a survival benefit and should be considered as an acceptable treatment for properly selected patients. Further investigation should focus on the prognostic factors that affect selection of patients who may benefit from an operative intervention.
